# Difficidin and bacilysin from *Bacillus amyloliquefaciens* FZB42 have antibacterial activity against *Xanthomonas oryzae* rice pathogens

**DOI:** 10.1038/srep12975

**Published:** 2015-08-13

**Authors:** Liming Wu, Huijun Wu, Lina Chen, Xinfang Yu, Rainer Borriss, Xuewen Gao

**Affiliations:** 1 Department of Plant Pathology, College of Plant Protection, Nanjing Agricultural University, Key Laboratory of Monitoring and Management of Crop Diseases and Pest Insects, Ministry of Education, Nanjing, China; 2 ABiTEP GmbH, Berlin, Germany

## Abstract

Bacterial blight and bacterial leaf streak are serious, economically damaging, diseases of rice caused by the bacteria *Xanthomonas oryzae* pv. *oryzae* and *X. oryzae* pv. *oryzicola*. *Bacillus amyloliquefaciens* FZB42 was shown to possess biocontrol activity against these *Xanthomonas* strains by producing the antibiotic compounds difficidin and bacilysin. Analyses using fluorescence, scanning electron and transmission electron microscopy revealed difficidin and bacilysin caused changes in the cell wall and structure of *Xanthomonas*. Biological control experiments on rice plants demonstrated the ability of difficidin and bacilysin to suppress disease. Difficidin and bacilysin caused downregulated expression of genes involved in *Xanthomonas* virulence, cell division, and protein and cell wall synthesis. Taken together, our results highlight the potential of *B. amyloliquefaciens* FZB42 as a biocontrol agent against bacterial diseases of rice, and the utility of difficidin and bacilysin as antimicrobial compounds.

The Gram-negative bacterial genus *Xanthomonas* can infect at least 350 different plants, resulting in significant economic losses in agriculture worldwide^1^,^2^,^3^. *Xanthomonas oryzae* pv. *oryzae* and *X. oryzae* pv. *oryzicola* are important rice pathogens, which cause bacterial rice blight and bacterial leaf streak of rice, respectively^2^. Traditional management practices, especially copper chemicals, increase the cost of production, leave residuals on crops and soil, and develop resistance in populations of target pathogens^3^,^4^. Therefore, there is a pressing need to develop cost-effective and convenient strategies that minimize environmental impact. Biological control agents, for example plant growth-promoting bacteria *Pseudomonas* and *Bacillus*^5^, have received a great deal of attention on account of being environmentally-friendly and versatile in their mode of action.

*Bacillus* spp. are attractive for use in farming systems because of their ability to form heat- and desiccation-resistant endospores which can survive the preparation of bacterial formulations^6^. *Bacillus amyloliquefaciens* FZB42 is the type strain for a group of plant-associated *Bacillus* spp. classified as *B. amyloliquefaciens* subsp. *plantarum*. *Bacillus amyloliquefaciens* FZB42 has the impressive ability to stimulate plant growth and to suppress plant pathogenic organisms, which distinguishes it from the related model organism *B. subtilis* 168, and has been commercially applied to a broad range of host plants^7^,^8^. The genome of strain FZB42 was sequenced and it harbors an array of giant gene clusters that produce several secondary metabolites with antimicrobial activity^9^. Its antifungal activity is attributed mainly to the nonribosomally synthesized cyclic lipopeptides bacillomycin D and fengycin^8^, its antibacterial activity is mainly due to non-ribosomal synthesis of polyketides^10^, its nematicidal activity is due to the ribosomally synthesized peptide antibiotic plantazolicin^11^, whilst its algicidal activity arises from the nonribosomal dipeptide bacilysin^12^.

In a previous study, we demonstrated that rice plants treated with *B. amyloliquefaciens* FZB42 suspensions showed significant improvement in resistance to *X. oryzae* pv. *oryzae* over untreated plants^13^. The aim of the present study was to identify the antibacterial substance(s) present in the culture suspensions of FZB42 and to gain insight into the underlying mechanisms responsible for the antagonistic effect against *Xanthomonas* spp. The results demonstrate that difficidin and bacilysin from *B. amyloliquefaciens* FZB42 have antibacterial activity against *X. oryzae* pv. *oryzae* and *X. oryzae* pv. *oryzicola*, and that cytotoxic effects cause apparent changes in the bacterial plasma membrane and structure.

## Results

### Difficidin and bacilysin have antibacterial activities against *X. oryzae* pv. *oryzae* and *X. oryzae* pv. *oryzicola*

**Figure 1:**
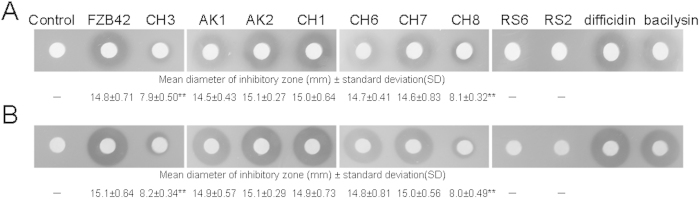
Detection of antagonistic action against *Xanthomonas oryzae* pv. *oryzae* (**A**) and *Xanthomonas oryzae* pv. *oryzicola* (**B**) by paper-disc agar diffusion assay. Bactericidal activity was tested as described in Methods. Control (Landy medium), FZB42 (wild type, producer of lipopeptides, polyketides and bacilysin), CH3 (Δ*sfp*::Em^r^, deficient in lipopeptide and polyketide synthesis), AK1 (Δ*bmyA*::Em^r^, deficient in bacillomycin D synthesis), AK2 (Δ*fenA*::Cm^r^, deficient in fengycin synthesis), CH1 (Δ*srfA*::Em^r^, deficient in surfactin synthesis), CH6 (Δ*bae*::Cm^r^, no synthesis of bacillaene), CH7 (Δ*mln*::Cm^r^, no synthesis of macrolactin), CH8 (Δ*dfn*::Em^r^, no synthesis of difficidin), RS6 (Δ*sfp*::Em^r^ Δ*bac*::Cm^r^, no lipopeptides, polyketides or bacilysin), and RS2 (Δ*bac*::Cm^r^ Δ*dfn*::Em^r^, deficient in bacilysin and difficidin). The diameters of inhibition zones (mm) included the paper disk diameter (5 mm). Data are expressed as means ± standard deviation (SD). − indicates no inhibitory activity. **indicates an extremely significant difference compared with FZB42 (P < 0.01).

To identify the active substances produced by *B. amyloliquefaciens* FZB42, we initially used a mutant strain devoid of non-ribosomal synthesis of lipopeptides and polyketides (strain CH3). In agar diffusion assays, strain CH3 resulted in a small zone of inhibition against *Xanthomonas*, and that zone was very significantly different (P < 0.01) from wild-type *B. amyloliquefaciens* FZB42, indicating that one or more lipopeptides and/or polyketides and/or other metabolites synthesized through the *sfp*-dependent pathway had the ability to suppress growth of *Xanthomonas* (Fig. 1).

To identify the anti-*Xanthomonas* substances, single mutants of *B. amyloliquefaciens* deficient in production of surfactin (CH1), bacillomycin D (AK1), fengycin (AK2), bacillaene (CH6), macrolactin (CH7) difficidin (CH8) and a double mutant (RS6), blocked in synthesis of lipopeptides and polyketides and production of bacilysin, were tested. Strains complemented for those genes were also examined. Agar diffusion tests illustrate that the inhibitory effect exerted by CH8 was clearly reduced relative to the wild-type (P < 0.01) and RS6 yielded no inhibition zone, whilst the corresponding complemented strains resulted in similar bactericidal effects to the wild-type, suggesting that difficidin and bacilysin act as antagonists of *X. oryzae* pv. *oryzae* and *X. oryzae* pv. *oryzicola* (Fig. 1, Fig. S2). This conclusion was corroborated by the absence of an antagonistic effect of strain RS2, which is devoid of difficidin and bacilysin, and efficient suppression of *Xanthomonas* by difficidin and bacilysin purified from FZB42 culture filtrates (Fig. 1).

### Effect of difficidin and bacilysin on viability of *Xanthomonas* spp. cells

We characterized *Xanthomonas* spp. cell development in the absence and presence of difficidin and bacilysin using phase contrast/fluorescence microscopy in combination with LIVE/DEAD BacLight bacterial viability staining (Figs S3 and S4; Table 1). Incubation of *Xanthomonas* spp. cell suspensions with the probes did not result in an increase in dead cells (red fluorescence) and, accordingly, the vast majority (95.58%, 96.09%) of the cell population remained alive (green fluorescence). In contrast, after 12 h of exposure to 10 μg/ml difficidin or bacilysin, the proportions of red fluorescent cells increased to 35.81%–40.48%. A large number of dead cells (86.67%–91.17%) were observed in the presence of 50 μg/ml difficidin or bacilysin as a consequence of the extensive accumulation of the red probe into the bacterial cells in response to membrane damage.

### Morphological and ultrastructural changes of *Xanthomonas* cells in the presence of difficidin and bacilysin

**Figure 2:**
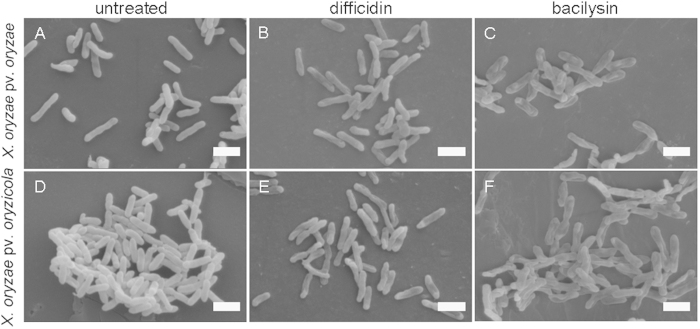
Morphological changes of *Xanthomonas* cells after exposure to 50 μg/ml difficidin or bacilysin for 12 h determined by SEM. (**A**) untreated *X. oryzae* pv. *oryzae*; (**B**) *X. oryzae* pv. *oryzae* treated with difficidin; (**C**) *X. oryzae* pv. *oryzae* treated with bacilysin; (**D**) untreated *X oryzae* pv. *oryzicola;* (**E**) *X. oryzae* pv. *oryzicola* treated with difficidin; (**F**) *X. oryzae* pv. *oryzicola* treated with bacilysin. Bars: 1 μm.

**Figure 3:**
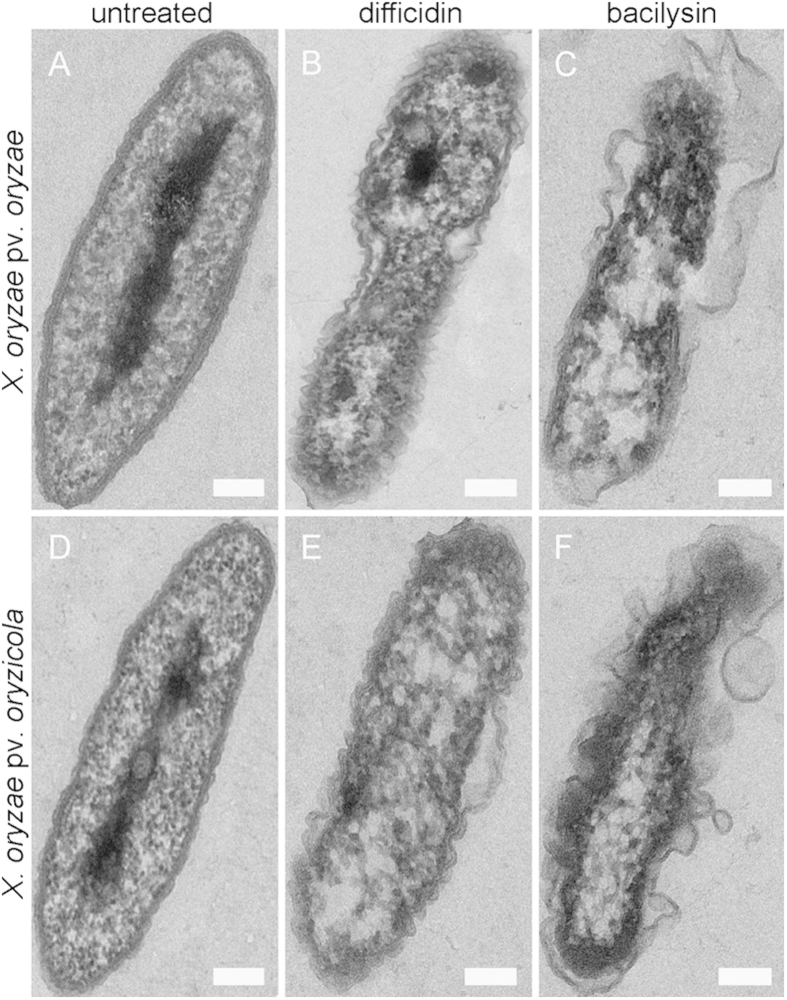
Ultrastructural effects of 50 μg/ml difficidin or bacilysin on *Xanthomonas* cells after 12 h determined by TEM. (**A**) an untreated *X. oryzae* pv. *oryzae* cell; (**B**) *X. oryzae* pv. *oryzae* treated with difficidin; (**C**) *X. oryzae* pv. *oryzae* treated with bacilysin; (**D**) an untreated *X. oryzae* pv. *oryzicola* cell; (**E**) *X. oryzae* pv. *oryzicola* treated with difficidin; (**F**) *X. oryzae* pv. *oryzicola* treated with bacilysin. Bars: 0.2 μm.

Visualization of the cellular damage caused to *X. oryzae* pv. *oryzae* and *X. oryzae* pv. *oryzicola* at the ultrastructural level by difficidin and bacilysin was undertaken by SEM and TEM analyses (Figs 2 and 3). In the SEM study, untreated control cells appeared intact, plump and typically rod-shaped with a smooth exterior (Fig. 2A,D). Upon exposure to difficidin or bacilysin, cell walls became loose and porous, distorted from their normal shape or even ruptured (Fig. 2B to C and Fig. 2E to F). By TEM, untreated cells showed a very distinct cell wall and a uniformly distributed electro-dense cytoplasm (Fig. 3A,D). After 12 h of treatment with difficidin or bacilysin, the lysis of the bacterial cell or a partial vesiculation of the membrane were clearly visible and resulted in plasmolysis and efflux of intracellular components. There were no evident electro-dense and basic structures (Fig. 3B to C and Fig. 3E to F). The damage due to bacilysin was more severe than that caused by difficidin.

### Biological control of rice diseases caused by *Xanthomonas* spp

**Figure 4:**
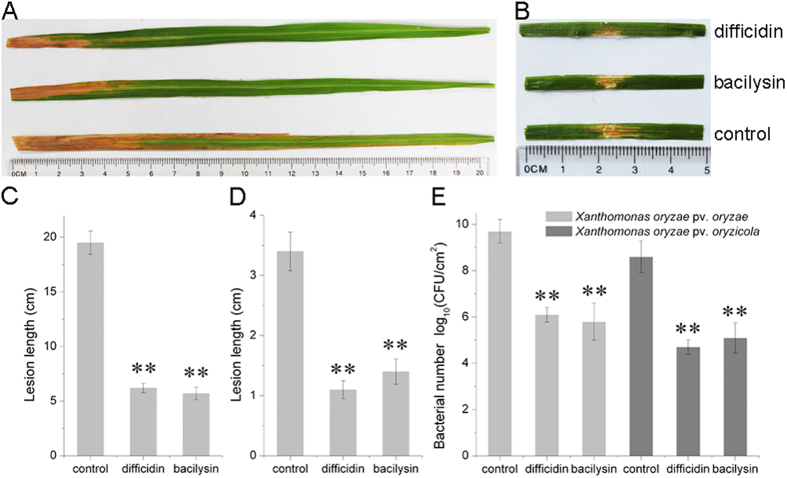
Pathogenicity test of *Xanthomonas oryzae* pv. *oryzae* and *Xanthomonas oryzae* pv. *oryzicola* strains on rice. (**A**) Representative result of lesion length symptom tests on the leaves of adult susceptible rice (cultivar 9311, two-month old) after treatment with 50 μg/ml difficidin and bacilysin, respectively. (**B**) Representative result of water-soaking lesion length tests on rice seedling leaves (cultivar 9311, two-week old) after infiltration with 50 μg/ml difficidin and bacilysin, respectively. (**C**) Calculated lesion lengths on the leaves of susceptible adult rice. (**D**) Calculated water-soaking lesion lengths on the leaves of rice seedlings. (**E**) The number of *Xanthomonas* cells in adult-susceptible rice leaves and rice seedling leaves after difficidin and bacilysin treatments. Data are expressed as means ± standard deviation (SD); **indicates an extremely significant difference compared with controls (P < 0.01).

To investigate the role of difficidin and bacilysin in resistance to bacterial leaf blight and bacterial leaf streak of rice, pathogenicity assays were performed. Figure 4 shows that rice plants (cultivar 9311) treated with difficidin and bacilysin exhibited a significant reduction in *Xanthomonas* virulence relative to controls. The lengths of lesions and the disease severities caused by the pathogens decreased remarkably. The protective rates for difficidin and bacilysin were 58.82%–72.31%. Simultaneously, the biocontrol efficacy of mutants against rice diseases caused by *Xanthomonas* spp. was also investigated. As previously observed in the *in vitro* assays, the mutants RS2 and RS6 impaired in the production of difficidin and bacilysin completely lost the ability to control bacterial leaf blight and bacterial leaf streak of rice, and leaves developed symptoms similar to those observed in untreated leaves (Fig. S5). The biocontrol abilities of the difficidin mutants CH3 and CH8 were slightly decreased compared with the wild-type strain (Fig. S5).

The population densities of *X. oryzae* pv. *oryzae* and *X. oryzae* pv. *oryzicola* on rice leaves were evaluated to associate the observed biocontrol activity with the antagonistic effect of difficidin and bacilysin on the number of phytopathogenic bacteria. A significant decrease of population levels, more than three orders of magnitude, was observed in comparison with controls when difficidin and bacilysin were applied before inoculation of rice with *Xanthomonas* spp. (Fig. 4E).

### Determination of *Xanthomonas* gene expression after exposure to difficidin and bacilysin

**Figure 5:**
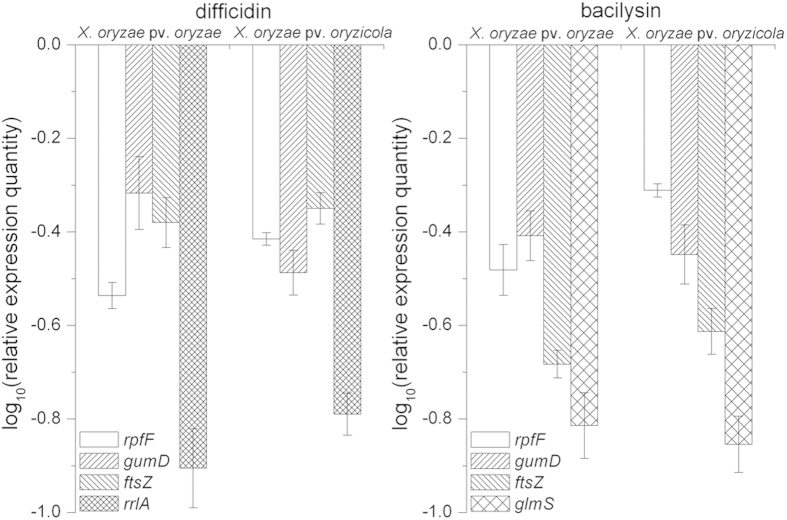
Quantitative real-time PCR analysis of expression of five genes (*rpfF*, *gumD*, *ftsZ*, *glmS*, *rrlA*) in *Xanthomonas* cells in response to difficidin and bacilysin treatment. Values were normalized to the levels of *16S rRNA*, an internal reference gene. The y-axis represents mean expression values ± SD relative to the control. The experiment was independently repeated five times.

Five genes (*rpfF*, *gumD*, *ftsZ*, *rrlA* and *glmS*) were chosen to explore the effects of difficidin and bacilysin on *Xanthomonas* gene expression. The *rpfF* gene is involved in production of a diffusible signal factor (DSF)^1^ and the *gumD* gene is a gene in the *gum* operon responsible for extracellular polysaccharide (EPS) biosynthesis^2^, which are both required for virulence. The *ftsZ* gene product is involved in cell division^4^. *rrlA*, a 23S rRNA gene, has been reported to be a binding site for macrolide antibiotics^14^,^15^. The *glmS* gene encodes glucosamine-6-phosphate synthase, which is important for the biosynthesis of peptidoglycan, a component of the bacterial cell wall^16^. Figure 5 shows that the transcriptional expression of *rpfF*, *gumD* and *ftsZ* were slightly downregulated, while the levels of *rrlA* and *glmS* decreased significantly, on treatment of *X. oryzae* pv. *oryzae* and *X. oryzae* pv. *oryzicola* with difficidin or bacilysin.

## Discussion

Numerous studies have demonstrated that biological control is an interesting and efficient strategy that might be applied in the management of plant diseases caused by the genus *Xanthomonas*. Zeriouh *et al*. (2011)^3^ reported that the iturin-like lipopeptides are essential components in the biological control arsenal of *B. subtilis* against cucurbit pathogenic bacteria *X. campestris* pv. *cucurbitae*. Silva *et al*. (2013)^4^ showed that alkyl gallates display a potent antibacterial activity against *X. citri* subsp. *citri*, the causal agent of Asiatic citrus canker. Wang *et al*. (2012)^17^ indicated that chitosan markedly inhibits the growth of pathogenic *Xanthomonas* isolated from *Euphorbia pulcherrima*. In the present study, we showed that difficidin and bacilysin from *B. amyloliquefaciens* FZB42 have antibacterial activities against *X. oryzae* pv. *oryzae* and *X. oryzae* pv. *oryzicola*.

Difficidin was first detected in fermentation broth of *B. subtilis* ATCC 39320 and characterized as a highly unsaturated 22-membered macrocylic polyene lactone phosphate ester^18^. The dipeptide bacilysin, consisting of a non-proteinogenic L-anticapsin and an N-terminal L-alanine, was first isolated by Foster and Woodruff from the soil bacterium *B. subtilis*^19^. Genome analysis of *B. amyloliquefaciens* FZB42 revealed gene clusters encoding the biosynthesis genes for difficidin and bacilysin, and these two substances were found in culture broths by HPLC^9^,^10^. The antibacterial activities of difficidin and bacilysin from *Bacillus* spp. have generally been described against medically important bacteria^19^,^20^; only a few reports have addressed their effects on plant pathogenic bacteria^21^. The results presented in this work show conclusively the crucial role of difficidin and bacilysin in the antagonistic effect against two important rice pathogens and their protective capability against rice diseases.

Study of a large number of macrolides revealed they could inhibit protein synthesis by binding to the large ribosomal subunit^22^,^23^. Canu *et al*. (2002)^14^ demonstrated that domains V and II of 23S rRNA (*rrl* gene) and proteins L22 and L4 are binding sites for macrolides. Difficidin, as one kind of macrolide, has been reported to rapidly inhibit protein synthesis and possibly also damage cell membranes^24^. Antimicrobial activity of bacilysin depends on the anticapsin moiety, which is released by peptidases. Intracellular anticapsin then blocks glucosamine synthetase, and, hence, bacterial peptidoglycan or fungal mannoprotein biosynthesis, resulting in protoplasting and lysis^12^,^19^,^25^. In this report, we found difficidin and bacilysin affected the cell wall, as evidenced by fluorescence and ultramicroscopic observations. The *rrlA* gene, the potential binding site of difficidin, and the *glmS* gene, the target for bacilysin, were downregulated significantly, as illustrated by qRT-PCR. This result was further confirmed by the reduction of *rrlA* expression detected using a transcriptional *lacZ* reporter, and of the glucosamine synthase activity encoded by *glmS* (Fig. S6). Moreover, the transcript levels of virulence genes *rpfF* and *gumD* decreased, which coincided with a decline in disease severities. A similar phenomenon was observed previously, in that bacilysin caused apparent changes in the algal cell wall and cell organelle membranes^12^.

In summary, our results support the view that difficidin and bacilysin are the main *X. oryzae* pv. *oryzae* and *X. oryzae* pv. *oryzicola* suppressing compounds in the culture filtrate of *B. amyloliquefaciens* FZB42. These strains are the causative agents of the important rice diseases bacterial blight and bacterial leaf streak, respectively. Since difficidin and bacilysin have not been previously used in agricultural management, this finding provides a potential option to use them or their producer strain FZB42 as an alternative to chemical bactericides to control rice diseases.

## Methods

### Bacterial strains and growth conditions

The bacterial strains used in this study are described in Table S1. *Bacillus* spp. were cultivated routinely on Luria broth (LB) medium solidified with 1.5% agar and fermented in Landy medium^21^,^26^. Nutrient agar (NA) medium^3^,^27^ was used to culture *X. oryzae* pv. *oryzae* and *X. oryzae* pv. *oryzicola*. When required, antibiotics were added to the following final concentrations: ampicillin 100 μg/ml, chloramphenicol 5 μg/ml, rifampicin 100 μg/ml and erythromycin 10 μg/ml.

### *In vitro* evaluation of antibacterial activity

The antibacterial activity of *B. amyloliquefaciens* cell-free culture filtrates was roughly analyzed as previously described^25^. The *B. amyloliquefaciens* strains were grown on Landy medium at 30°C with agitation for 38 h. After centrifugation at 12,000 × g for 10 min, the supernatants were filtered through 0.22 μm Millipore membranes. Five microliters of culture filtrate obtained after centrifugation and filtration were applied to a paper disk (5 mm diameter) placed on NA agar inoculated with *X. oryzae* pv. *oryzae* or *X. oryzae* pv. *oryzicola*. Landy medium was used instead of culture supernatant as the control. The plates were incubated at 28 °C for 48 h and the inhibition zones (mm) included the paper disk diameter.

### Purification of difficidin and bacilysin

To purify difficidin, culture filtrates from *B. amyloliquefaciens* FZB42 grown in Landy medium were absorbed onto an amberlite XAD16 column which was washed with distilled water and eluted with 100% methanol. The eluate was lyophilized and dissolved in methanol containing 10% distilled water. High-performance liquid chromatography-electrospray ionization (HPLC-ESI) of difficidin was performed essentially as previously described^10^,^21^. The retention time of difficidin was 8.574 min as detected by absorbance at 280 nm and the expected molecular mass of 544 Da. Eluate at the corresponding retention time was collected and lyophilized to obtain pure difficidin (Fig. S1). Pure bacilysin was produced as in Wu *et al*. (2014)^12^.

### LIVE/DEAD BacLight bacterial viability staining

The viability assay was performed using the LIVE/DEAD BacLight bacterial viability staining kit L7012 (Invitrogen, Molecular Probes, USA) as previously described^3^. The kit consists of two colored fluorescence stains: a green-fluorescent SYTO 9 stain and a red-fluorescent propidium iodide (PI) stain. When used in an appropriate mixture, live bacteria with intact membranes fluoresce green, while bacteria with damaged membranes fluoresce red. *Xanthomonas* cells treated with difficidin or bacilysin (10 μg/ml, 50 μg/ml) for 12 h were centrifuged at 1000 × g for 10 min, and resuspended in 10 mM sodium phosphate buffer (pH 7.4) at a concentration of 10^7^–10^8^ cells/ml. Then, 10 μl of the molecular probes, prepared as recommended by the manufacturer, were added, and the cell suspensions were incubated for 15 min at 25 °C in the dark. The samples were analyzed by an Olympus BX43 microscope using cellSens Standard Software (Tokyo, Japan).

### SEM and TEM studies

Scanning electron microscopy (SEM) and transmission electron microscopy (TEM) analysis were used to determine the effects of difficidin and bacilysin on *Xanthomonas* cells at the ultrastructural level. *X. oryzae* pv. *oryzae* and *X. oryzae* pv. *oryzicola* treated with 50 μg/ml difficidin or bacilysin were centrifuged and prefixed with 2.5% glutaraldehyde. Fixed cells were rinsed three times for 10 minutes with 100 mM phosphate buffer, postfixed for 3 h in 1% osmium tetroxide, and dehydrated through an ethanol gradient. For SEM analysis, samples were coated with gold and analyzed on a Hitachi S-3000N scanning electron microscope (Hitachi, Japan). For TEM analysis, samples were embedded in Epon 812, sectioned with an ultramicrotome and examined under a Hitachi H-600 transmission electron microscope.

### Pathogenicity test in rice plants

Pathogenicity assays were conducted in a glasshouse at 25−28 °C as previously described^2^,^13^. In brief, *X. oryzae* pv. *oryzae* and *X. oryzae* pv. *oryzicola* strains were cultivated in NA broth at 28 °C with appropriate antibiotics. Two days before inoculation with the bacterial pathogen, rice (cultivar 9311) leaves were sprayed with 50 μg/ml difficidin, bacilysin, or water as the control. For observation of lesion length due to *X*. *oryzae* pv. *oryzae*, two-month-old rice plants were inoculated with a suspension of 10^8^ CFU (colony forming unit)/ml of strain PXO99A by the leaf-clipping method. For observation of water-soaking due to *X*. *oryzae* pv. *oryzicola*, a suspension (10^8^ CFU/ml) of strain RS105 was infiltrated into the leaves of two-week old rice seedlings by needleless syringe. The disease symptoms were recorded after 15 days of incubation and the protective rate was calculated by using the following equation: protective rate (%) = (1 − T/C) × 100, where T (treatment) and C (control) are lesion lengths with and without treatment, respectively. Population levels of *X. oryzae* pv. *oryzae* and *X. oryzae* pv. *oryzicola* in leaf tissue were estimated by serial dilutions and colony counts on plates of selective medium after 2 days of incubation at 28 °C.

### Quantitative real time-PCR analysis

For the determination of gene expression, *Xanthomonas* spp. were exposed to 50 μg/ml difficidin or bacilysin for 2 h, respectively. Total RNA was extracted using a Bacterial RNA Kit (Omega Bio-Tek, USA) according to the manufacturer’s instructions. First-strand cDNA was synthesized using Reverse Transcriptase (TaKaRa Bio Inc, Dalian, China) with random hexamer primers and the resulting cDNA was used as the template for subsequent PCR amplification. qRT-PCR was performed with SYBR Premix Ex Taq (TaKaRa Bio Inc, Dalian, China) using a 7500 Fast Real-Time PCR Detection System. Gene *16S rRNA* was used as the internal reference for normalization. Primers for these genes are listed in Table S2.

### Statistical analysis

Each experiment was independently repeated at least three times. Data were analyzed using analysis of variance, followed by a Fisher least significant difference test; the statistics software SPSS v16.0 (SPSS Inc., Chicago, USA) was employed^26^,^27^.

## Additional Information

**How to cite this article**: Wu, L. *et al*. Difficidin and bacilysin from *Bacillus amyloliquefaciens* FZB42 have antibacterial activity against *Xanthomonas oryzae* rice pathogens. *Sci. Rep*. **5**, 12975; doi: 10.1038/srep12975 (2015).

## Supplementary Material

Supplementary Information

## Figures and Tables

**Table 1:** Quantification of the viability of *Xanthomonas* cells after exposure to difficidin and bacilysin.

Treatment		*Xanthomonas oryzae pv. oryzae*		*Xanthomonas oryzae pv. oryzicola*	
		live cells (%)	dead cells (%)	live cells (%)	dead cells (%)
untreated		96.09	3.91	95.58	4.42
difficidin	10 μg/ml	59.52	40.48	62.85	37.15
	50 μg/ml	8.83	91.17	10.28	89.72
bacilysin	10 μg/ml	63.65	36.35	64.19	35.81
	50 μg/ml	11.25	88.75	13.33	86.67

The number of total, live (green) and dead (red) cells was counted in ten different microscopic fields. The values indicate the percentages of live and dead cells in the suspensions.

## References

[b1] Chatterjee S. & Sonti R. V. *rpfF* mutants of *Xanthomonas oryzae* pv. *oryzae* are deficient for virulence and growth under low iron conditions. Mol. Plant Microbe. Interact. 15, 463–471 (2002). 12036277 10.1094/MPMI.2002.15.5.463

[b2] Qian G. . Proteomic analysis reveals novel extracellular virulence-associated proteins and functions regulated by the diffusible signal factor (DSF) in *Xanthomonas oryzae pv. oryzicola*. J. Proteome Res. 12, 3327–3341 (2013). 23688240 10.1021/pr4001543

[b3] Zeriouh H. . The iturin-like lipopeptides are essential components in the biological control arsenal of *Bacillus subtilis* against bacterial diseases of cucurbits. Mol Plant Microbe Interact. 24, 1540–1552 (2011). 22066902 10.1094/MPMI-06-11-0162

[b4] Silva I. C. . Antibacterial activity of alkyl gallates against *Xanthomonas citri* subsp. *citri*. J Bacteriol. 195, 85–94 (2013). 23104804 10.1128/JB.01442-12 PMC3536167

[b5] de Oliveira A. G. . Evaluation of the antibiotic activity of extracellular compounds produced by the *Pseudomonas* strain against the *Xanthomonas citri* pv. *citri* 306 strain. Bio Control. 56, 125–131 (2011).

[b6] Chowdhury S. P. . Effects of *Bacillus amyloliquefaciens* FZB42 on lettuce growth and Health under Pathogen Pressure and Its Impact on the Rhizosphere Bacterial Community. PLoS One. 8, e68818, 10.1371/journal.pone.0068818 (2013). 23935892 PMC3720850

[b7] Fan B. . Efficient colonization of plant roots by the plant growth promoting bacterium *Bacillus amyloliquefaciens* FZB42, engineered to express green fluorescent protein. J Biotechnol. 151, 303–311 (2011). 21237217 10.1016/j.jbiotec.2010.12.022

[b8] Koumoutsi A. . Structural and Functional Characterization of Gene Clusters Directing Nonribosomal Synthesis of Bioactive Cyclic Lipopeptides in *Bacillus amyloliquefaciens* Strain FZB42. J Bacteriol. 186, 1084–1096 (2004). 14762003 10.1128/JB.186.4.1084-1096.2004 PMC344220

[b9] Chen X. H. . Genome analysis of *Bacillus amyloliquefaciens* FZB42 reveals its potential for biocontrol of plant pathogens. J Biotechnol. 140, 27–37 (2009). 19041913 10.1016/j.jbiotec.2008.10.011

[b10] Chen X. H. . Structural and functional characterization of three polyketide synthase gene clusters in *Bacillus amyloliquefaciens* FZB 42. J Bacteriol. 188, 4024–4036 (2006). 16707694 10.1128/JB.00052-06 PMC1482889

[b11] Liu Z. . The highly modified microcin peptide plantazolicin is associated with nematicidal activity of *Bacillus amyloliquefaciens* FZB42. Appl Microbiol Biotechnol. 97, 10081–10090 (2013). 24085393 10.1007/s00253-013-5247-5

[b12] Wu L. . Bacilysin from *Bacillus amyloliquefaciens* FZB42 has specific bactericidal activity against harmful algal bloom specie. Appl Environ Microbiol. 80, 7512–7520 (2014). 25261512 10.1128/AEM.02605-14 PMC4249228

[b13] Wang W. . Comparative proteomic analysis of rice seedlings in response to inoculation with *Bacillus cereus*. Lett Appl Microbiol. 56, 208–215 (2013). 23216197 10.1111/lam.12035

[b14] Canu A. . Diversity of ribosomal mutations conferring resistance to macrolides, clindamycin, streptogramin, and telithromycin in *Streptococcus pneumoniae*. Antimicrob Agents Chemother. 46, 125–131 (2002). 11751122 10.1128/AAC.46.1.125-131.2002 PMC126998

[b15] Gregory S. T., Cate J. H. & Dahlberg A. E. Spontaneous erythromycin resistance mutation in a 23S rRNA gene, *rrlA*, of the extreme thermophile *Thermus thermophilus* IB-21. J Bacteriol. 183, 4382–4385 (2001). 11418580 10.1128/JB.183.14.4382-4385.2001 PMC95329

[b16] Wojciechowski M., Milewski S., Mazerski J. & Borowski E. Glucosamine-6-phosphate synthase, a novel target for antifungal agents. Molecular modelling studies in drug design. Acta Biochim. Pol. 52, 647–653 (2005). 16082410

[b17] Wang Y. . Action of chitosan against *Xanthomonas* pathogenic bacteria isolated from *Euphorbia pulcherrima*. Molecules 17, 7028–7041 (2012). 22678416 10.3390/molecules17067028 PMC6268448

[b18] Wilson K. E. . Difficidin and oxydifficidin: novel broad spectrum antibacterial antibiotics produced by *Bacillus subtilis*. II. Isolation and physico-chemical characterization. J Antibiot. 40, 1682–1691 (1987). 3429336 10.7164/antibiotics.40.1682

[b19] Kenig M. & Abraham E. P. Antimicrobial activities and antagonists of bacilysin and anticapsin. J Gen Microbiol. 94, 37–45 (1976). 819623 10.1099/00221287-94-1-37

[b20] Zimmerman S. B. . Difficidin and oxydifficidin: novel broad spectrum antibacterial antibiotics produced by *Bacillus subtilis*. I. Production, taxonomy and antibacterial activity. J Antibiot. 40, 1677–1681 (1987). 3123448 10.7164/antibiotics.40.1677

[b21] Chen X. H. . Difficidin and bacilysin produced by plant-associated *Bacillus amyloliquefaciens* are efficient in controlling fire blight disease. J Biotechnol. 140, 38–44 (2009). 19061923 10.1016/j.jbiotec.2008.10.015

[b22] Wilson D. N. Ribosome-targeting antibiotics and mechanisms of bacterial resistance. Nat Rev Microbiol. 12, 35–48 (2014). 24336183 10.1038/nrmicro3155

[b23] Krokidis M. G. . Insights into the mode of action of novel fluoroketolides, potent inhibitors of bacterial protein synthesis. Antimicrob Agents Chemother. 58, 472–480 (2014). 24189263 10.1128/AAC.01994-13 PMC3910732

[b24] Zweerink M. M. & Edison A. Difficidin and oxydifficidin: novel broad spectrum antibacterial antibiotics produced by *Bacillus subtilis*. III. Mode of action of difficidin. J Antibiot. 40, 1692–1697 (1987). 2448279 10.7164/antibiotics.40.1692

[b25] Wu L. . Bacilysin overproduction in *Bacillus amyloliquefaciens* FZB42 markerless derivative strains FZBREP and FZBSPA enhances antibacterial activity. Appl Microbiol Biotechnol. 99, 4255–4263 (2015). 25472439 10.1007/s00253-014-6251-0

[b26] Chen X. H., Koumoutsi A., Scholz R. & Borriss R. More than anticipated - production of antibiotics and other secondary metabolites by *Bacillus amyloliquefaciens* FZB42. J Mol Microbiol Biotechnol. 16, 14–24 (2009). 18957859 10.1159/000142891

[b27] Salzberg S. L. . Genome sequence and rapid evolution of the rice pathogen *Xanthomonas oryzae* pv. *oryzae* PXO99^A^. BMC genomics 9, 204, 10.1186/1471-2164-9-204 (2008). 18452608 PMC2432079

