# Supplementary material for: Difficidin and bacilysin from Bacillus amyloliquefaciens FZB42 have antibacterial activity against Xanthomonas oryzae rice pathogens
Source: Sci Rep. 2015 Aug 13;5:12975. doi: 10.1038/srep12975 (PMC4534799; doi:10.1038/srep12975)
Supplement: Supplementary Information [file srep12975-s1.pdf]

Supplementary Information

Difficidin and bacilysin from *Bacillus amyloliquefaciens* FZB42 have antibacterial activity against *Xanthomonas oryzae* rice pathogens

Liming Wu[1], Huijun Wu[1], Lina Chen[1], Xinfang Yu[1], Rainer Borriss[2], Xuewen Gao[1 *]

[1]Department of Plant Pathology, College of Plant Protection, Nanjing Agricultural University, Key Laboratory of Monitoring and Management of Crop Diseases and Pest Insects, Ministry of Education, Nanjing, China

[2]ABiTEP GmbH, Berlin, Germany

[*] Corresponding author

Corresponding author:

Xuewen Gao

Email: gaoxw@njau.edu.cn

Telephone/Fax: 86-25-84395268

Table S1. Bacterial strains used in this study.

Strain	Description	Source or reference
B. amyloliquefaciens		
FZB42	Wild type, producer of lipopeptides, polyketides and bacilysin	8
AK1	△*bmyA*::Em^r, deficient in bacillomycin D synthesis	8
AK2	△*fenA*::Cm^r, deficient in fengycin synthesis	8
CH1	△*srfA*::Em^r, deficient in surfactin synthesis	8
CH3	△*sfp*::Em^r, deficient in lipopeptides and polyketides synthesis	10
CH6	△*bae*::Cm^r, no synthesis of bacillaene	10
CH7	△*mln*::Cm^r, no synthesis of macrolactin	10
CH8	△*dfn*::Em^r, no synthesis of difficidin	10
RS2	△*bac*::Cm^r △*dfn*::Em^r, deficient in bacilysin and difficidin	26
RS6	△*sfp*::Em^r △*bac*::Cm^r, no lipopeptides, polyketides and bacilysin	21
Rice pathogenic bacteria		
PXO99[A]	*Xanthomonas oryzae* pv. *oryzae*, causal agent of bacterial blight	27
RS105	*Xanthomonas oryzae* pv. *oryzicola*, causal agent of bacterial leaf streak, Rif^r	2

Table S2. DNA primers used in this study.

Xanthomonas oryzae* pv. *oryzae		***Xanthomonas oryzae* pv. *oryzicola***	
Name	Sequence (5´-3´)	Name	Sequence (5´-3´)
16S-F	TGGCAACTAAGGACAAGGG	16S-F	AATGGGCGCAAGCCTGATC
16S-R	AAGCGGTGGAGTATGTGG	16S-R	AACCACCACCTACGCACGC
rpfF-F	GTTGGTCTGATAGCGGGTGA	rpfF-F	TAGCGGGTGATGTCGTCG
rpfF-R	TGAAGAACCGCAGCGTGAG	rpfF-R	TGAAGAACCGCAGCGTGAG
gumD-F	TTTCATAGTGCGTGTTGTGCT	gumD-F	AACGACGCACGCATCACG
gumD-R	AACGACGCACGCATCACG	gumD-R	ATCCAGCCACAGCGACCAAC
ftsZ-F	CTGCTGGACGATGTGAACCTG	ftsZ-F	CCGTGATGTCGGAAATGGG
ftsZ-R	ATAATCGTTGGGCAGGTCAGC	ftsZ-R	GCCAGGTTCACATCGTCCAG
glmS-F	CCCCAGCGATTTGGCGTAC	glmS-F	ACGATCAGATGCTGTGGGTTC
glmS-R	GCGTATGCGAACCCACAGC	glmS-R	GCCAAGGGTTTCCCGTCAT
rrlA-F	ACAGGGTGGTATTTCAAGG	rrlA-F	GCAAACTCCGAATACCAG
rrlA-R	GCCGCTGCGACTGTTTAT	rrlA-R	CAACCTCCTGGCTGTCTAT

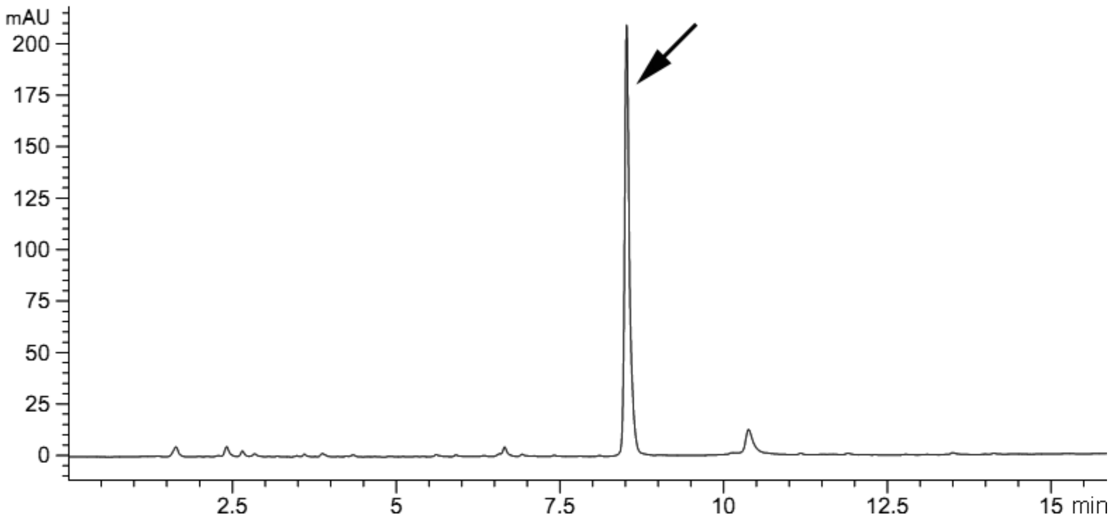

Fig. S1. HPLC analysis of difficidin from *B. amyloliquefaciens* FZB42. The retention time of difficidin is 8.574 min

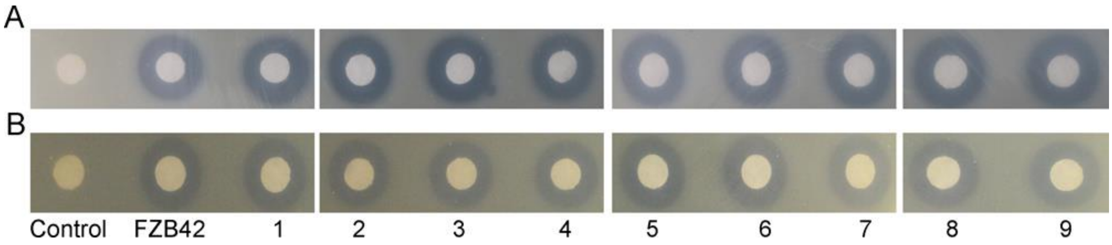

Fig. S2. Detection of antagonistic action of *B. amyloliquefaciens* mutants strains against *Xanthomonas oryzae* pv. *oryzae* (A) and *Xanthomonas oryzae* pv. *oryzicola* (B) by paper-disc agar diffusion assay. 1, complemented FZB42△*sfp*△*amyE*::*sfp*; 2, complemented FZB42△*bmyA*△*amyE*::*bmyA*; 3, complemented FZB42△*fenA*△*amyE*::*fenA*; 4, complemented FZB42△*srfA*△*amyE*::*srfA*; 5, complemented FZB42△*bae*△*amyE*::*bae*; 6, complemented FZB42△*mln*△*amyE*::*mln*; 7, complemented FZB42△*dfn*△*amyE*::*dfn*; 8, complementation of RS6; 9, complementation of RS2.

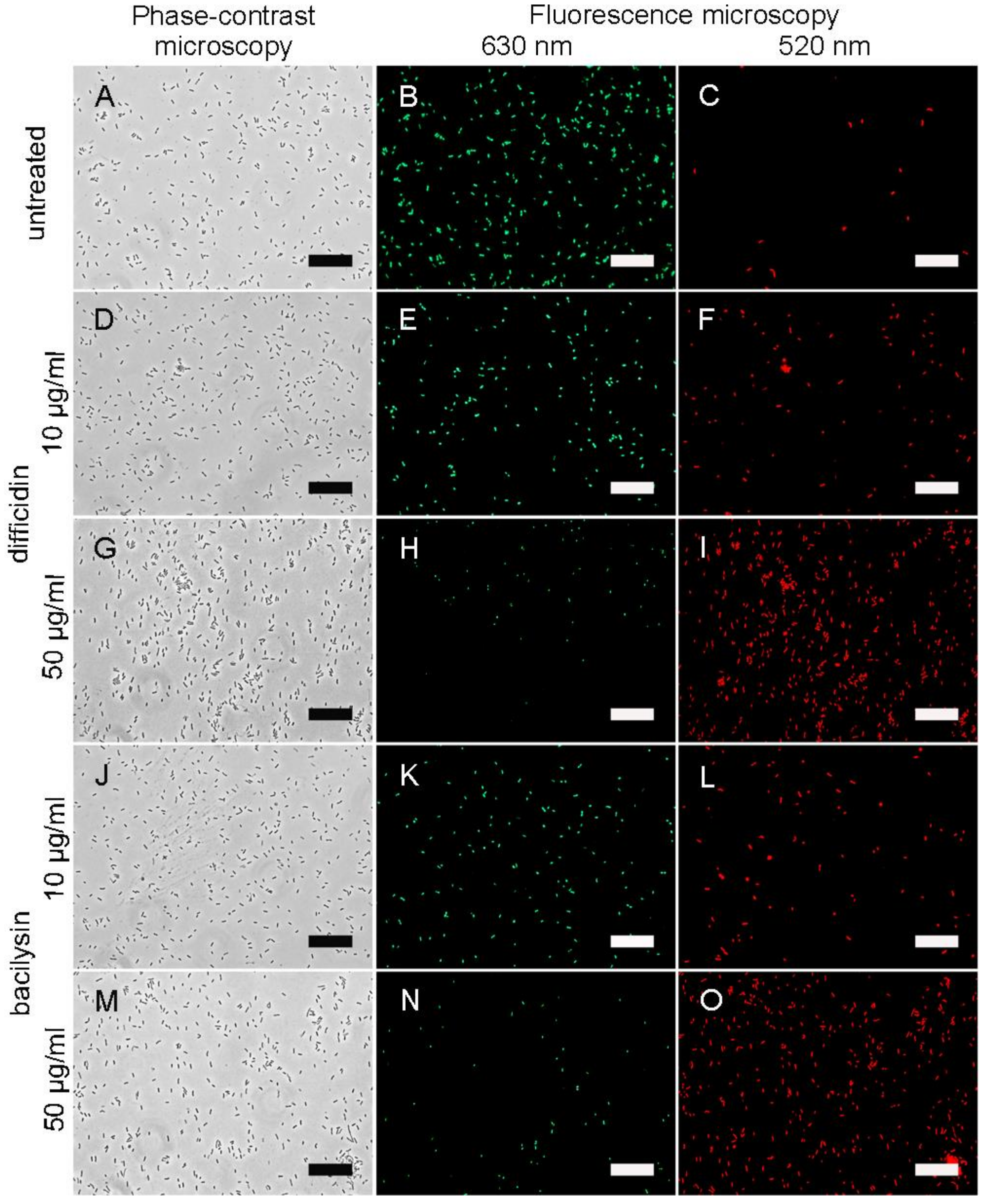

Fig. S3. Phase-contrast and fluorescence microscope images of *Xanthomonas oryzae* pv. *oryzae* cells in the absence and presence of difficidin or bacilysin after 12 h of cultivation. Staining using the LIVE/DEAD BacLight bacterial viability kit assessed bacterial viability: green areas indicate live cells; red areas indicate dead cells. Untreated *X. oryzae* pv. *oryzae* cells were observed by A, phase-contrast microscopy and B-C, fluorescence microscopy. *X. oryzae* pv. *oryzae* cells after treatments with difficidin (10 μg/ml, 50 μg/ml) were examined by D and G, phase-contrast microscopy and E-F and H-I, fluorescence microscopy. Pictures were taken after treatments with bacilysin (10 μg/ml, 50 μg/ml) from the J to L and M to O. Bars indicate 20 μm.

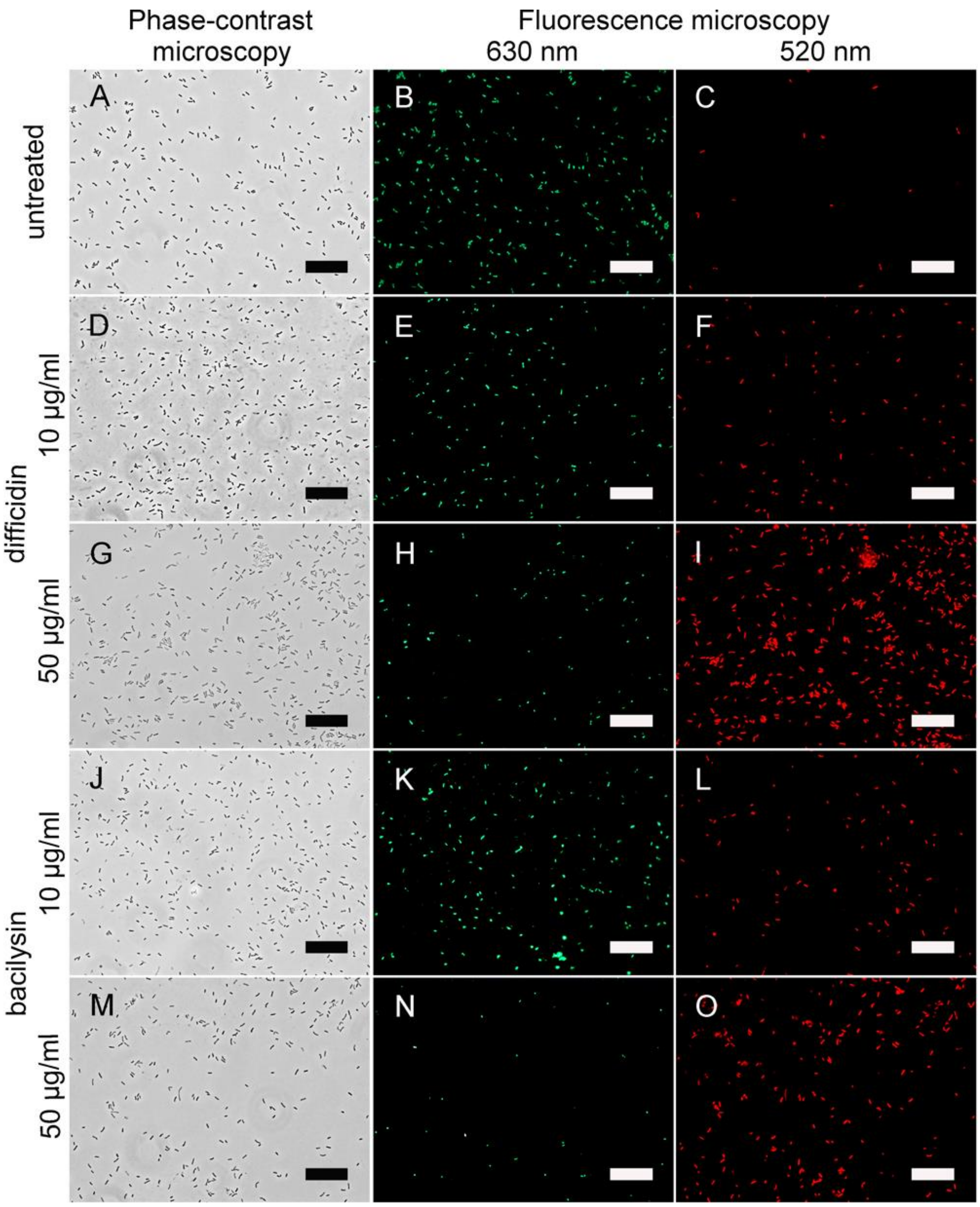

Fig. S4. The phase-contrast and fluorescence microscope images of *Xanthomonas oryzae* pv. *oryzicola* cells in the absence and presence of difficidin and bacilysin after 12 h of cultivation. Staining using the LIVE/DEAD BacLight bacterial viability kit assessed bacterial viability: green areas indicate live cells; red areas indicate dead cells. Bars: 20 μm.

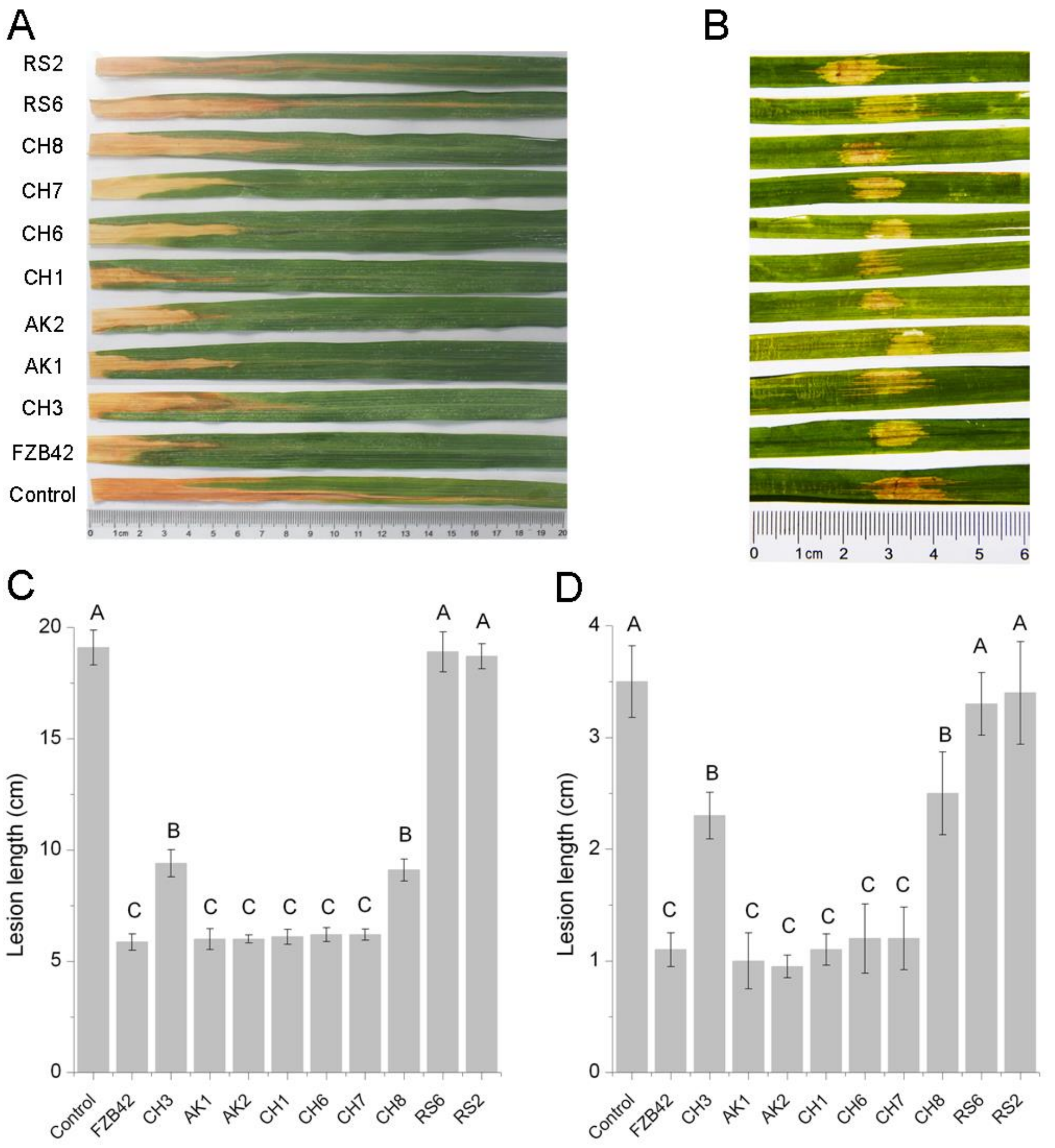

Fig. S5. Pathogenicity test of *Xanthomonas oryzae* pv. *oryzae* and *Xanthomonas oryzae* pv. *oryzicola* strains on rice. A. Representative result of lesion length symptom tests on the leaves of adult susceptible rice after treatment with *B. amyloliquefaciens* FZB42 mutants. B. Representative result of water-soaking lesion length tests on rice seedling leaves after infiltration with *B. amyloliquefaciens* FZB42 mutants. C. Calculated lesion lengths on the leaves of susceptible adult rice. D. Calculated water-soaking lesion lengths on the leaves of rice seedlings. Data are expressed as means ± standard deviation (SD); Different letters indicate means that differ statistically at $P < 0.01$.

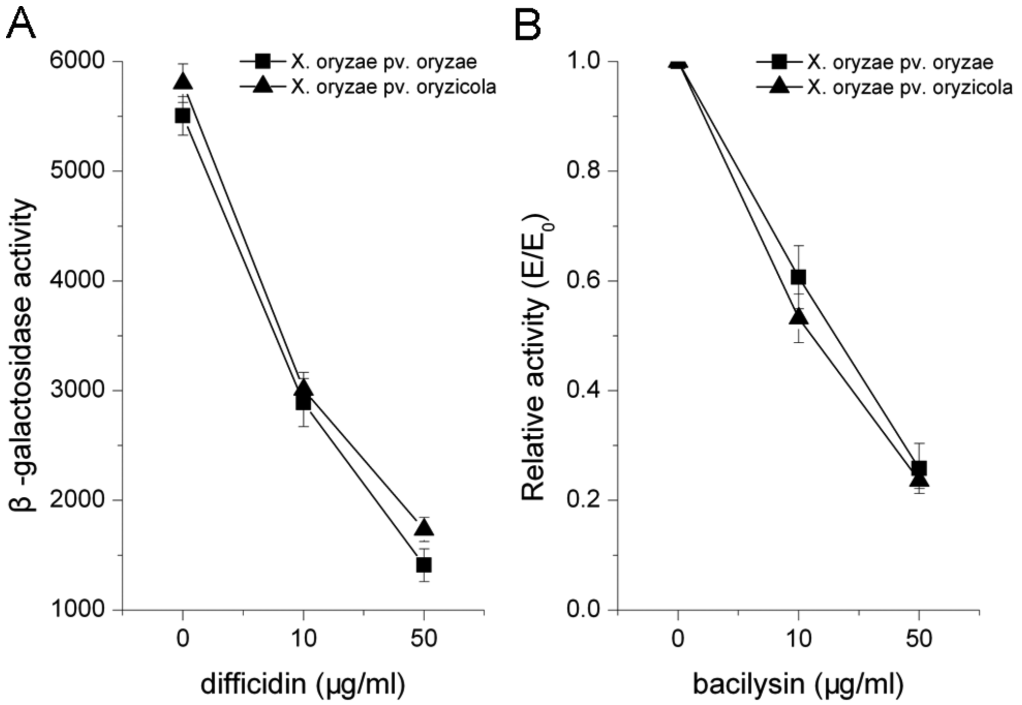

Fig. S6. The level of gene *rrlA* and of glucosamine synthase activity (encoded by *glmS*) in *Xanthomonas* spp. A. The *rrlA* activity in *Xanthomonas* spp. treated with 10 μg/ml and 50 μg/ml difficidin for 30 min. β-galactosidase activity was assayed using o-nitrophenyl-β-D-galactopyranoside as the substrate and is reported in Miller units. B. The glucosamine synthase activity of *Xanthomonas* spp. treated with 10 μg/ml and 50 μg/ml bacilysin for 30 min. The measured activity in the sample containing no bacilysin (E_0) was 0.501 units for *X. oryzae* pv. *oryzae* and 0.489 units for *X. oryzae* pv. *oryzicola*. E represents the corresponding relative residual activities in the presence of bacilysin.
